# Antibodies Against the C-Terminus of ApoA-1 Are Inversely Associated with Cholesterol Efflux Capacity and HDL Metabolism in Subjects with and without Type 2 Diabetes Mellitus

**DOI:** 10.3390/ijms20030732

**Published:** 2019-02-09

**Authors:** Robin P. F. Dullaart, Sabrina Pagano, Frank G. Perton, Nicolas Vuilleumier

**Affiliations:** 1 Department of Endocrinology, University Medical Center Groningen, University of Groningen, 9713 GZ Groningen, The Netherlands; f.g.perton@umcg.nl; 2 Division of Laboratory Medicine, Department of Diagnostics, Hộpitaux Universitaires de Geneve, 1211 Geneva, Switzerland; Sabrina.Pagano@hcuge.ch (S.P.); Nicolas.Vuilleumier@hchuge.nl (N.V.); 3 Department of Internal Medicine Specialities, Medical Faculty, Hộpitaux Universitaires de Geneve, 1211 Geneva, Switzerland

**Keywords:** anti-apoA-1 autoantibodies, cholesterol efflux capacity, cholesterol esterification, cholesteryl ester transfer, high-density lipoproteins, Type 2 diabetes mellitus

## Abstract

Background: We determined relationships of cholesterol efflux capacity (CEC), plasma cholesterol esterification (EST) and cholesteryl ester transfer (CET) with anti-c-terminus apoA-1 (Ac-terAA1) and anti-apolipoprotein (apo)-1 (AAA1) autoantibodies in subjects with and without Type 2 diabetes mellitus (T2D). Methods: In 75 T2D subjects and 75 nondiabetic subjects, Ac-terAA1 and AAA1 plasma levels were measured by enzyme-linked immunosorbent assay. CEC was measured as [^3^H]-cholesterol efflux from human cultured fibroblasts to diluted individual subject plasma. Plasma EST and CET were assayed by isotope methods. Results: Ac-terAA1 and AAA1 levels and were similar between T2D and control subjects. Univariate regression analysis (*n* = 150) demonstrated that Ac-terAA1 levels were inversely correlated with CEC, EST, CET, total cholesterol, non-HDL cholesterol, triglycerides and apolipoprotein B, (*p* < 0.05 to *p* < 0.01), but not with glucose and HbA1c. In separate multivariable linear regression models, CEC, EST and CET were inversely associated with Ac-terAA1 levels independently of age, sex, T2D and drug use (β = −0.186, *p* = 0.026; β = −0.261, *p* < 0.001; and β = −0.321, *p* < 0.001; respectively). These associations were lost after additional adjustment for non-HDL cholesterol and triglycerides. No associations were observed for AAA1. Conclusions: CEC, plasma EST and CET are inversely associated with Ac-terAA1 autoantibodies, conceivably attributable to an inverse relationship of these autoantibodies with apolipoprotein B-containing lipoproteins.

## 1. Introduction

During the past few years, evidence has accumulated that autoantibodies against high-density lipoproteins (HDL) and its components may impact on atherothrombotic processes that play a role in the pathogenesis of cardiovascular disease (CVD) [1]. Immunological assays to detect such antibodies have been recently developed in several laboratories and are currently available for clinical use [2,3]. Autoantibodies of IgG subclass against apolipoprotein A-1 (AAA1), the major apolipoprotein of HDL, have been shown to be elevated in subjects with established CVD, to be associated with worse outcome after acute myocardial infarction and stroke, and to predict incident atherosclerotic CVD and overall mortality in the general population [4,5,6,7,8,9]. Furthermore, AAA1 were shown to promote sterile inflammation in vitro and in vivo, accelerating the development of atherosclerosis and atherothrombosis in mice [10,11,12,13]. In humans, the polyclonal AAA1 response has been shown to be oriented against the last alpha helix of the c-terminus part of apolipoprotein A-1 (apoA-1) (amino acids: 220–242) [14,15], and the corresponding mimetic peptide can used for both the detection of anti-C-terminus apoA-1 (Ac-terAA1) and the neutralization of AAA1 deleterious effect in vitro [15].

Inverse associations have been observed between AAA1 levels and total cholesterol, low-density lipoprotein cholesterol and HDL cholesterol [7,13]. These autoantibodies could, therefore, interfere with cholesterol metabolism on top of their established proinflammatory and prothrombotic properties. Cellular efflux of cholesterol to extracellular acceptors (cholesterol efflux capacity (CEC)) provides the initial step in the reverse cholesterol transport (RCT) pathway, whereby cholesterol is transported back from the arterial wall to the liver for metabolism and excretion in the bile [16,17,18,19,20]. CEC represents a key metric of HDL function, and it has been suggested that impaired CEC represents an important predictor of CVD [19,20]. HDL metabolism is a complex process regulated by a number of interdependent pathways, starting with the generation of small lipid-poor HDL particles, i.e., pre-β-HDL, followed by esterification of free cholesterol (EST), which results in HDL maturation, and subsequent cholesteryl ester transfer (CET) to apolipoprotein B (apoB)-containing lipoproteins [21,22]. AAA1 and anti-HDL antibodies have been shown to impair the antioxidant function of HDL through paraoxonase-1 inhibition [3,23,24,25]. Such an effect on HDL function underscores the relevance of testing the hypothesis that AAA1 and Ac-terAA1 may interfere with CEC and HDL metabolism. Anti-apoA-1 autoantibodies have been found to be elevated in patients with Type 2 diabetes mellitus (T2D), though particularly only in those with CVD [26]. T2D is characterized by increased plasma EST and CET [27,28,29], which makes the diabetic state a relevant condition for which to interrogate the impact of AAA1 and Ac-terAA1 on plasma EST and CET. No data are currently available concerning the association of apoA1 autoantibodies with CEC and other metrics of HDL metabolism.

The present study was, therefore, initiated to delineate relationships of CEC, plasma EST and CET with AAA1 and Ac-terAA1. Furthermore, we explored the possible impact of T2D on such associations in view of abnormalities in HDL metabolism in this condition [27,28,29].

## 2. Results

The study population consisted of 75 control subjects and 75 T2D patients (Table 1). Seventeen diabetic patients were taking sulfonylurea alone and 15 were taking metformin alone, whereas both drugs were used by 21 patients. Antihypertensive medication (in most cases, angiotensin-converting enzyme inhibitors, angiotensin II receptor antagonists and diuretics, alone or in combination) was used by 29 T2D patients, but not in control subjects (*p* < 0.001). Oral contraceptives were taken by 4 nondiabetic women. Diabetic patients were older, more obese, had higher blood pressure and higher fasting glucose and HbA1c levels than control subjects (Table 1). HDL cholesterol and apoA-1 were lower in diabetic patients, coinciding with higher triglycerides. Pre-β-HDL formation was not different between the groups, but phospholipid transfer protein (PLTP) activity, lecithin–cholesterol acylesterase (LCAT) activity, cholesteryl ester transfer protein (CETP) mass, EST and CET were increased in T2D patients. CEC was not different between diabetic and control subjects (Table 1). Median AAA1 and Ac-terAA1 levels were similar between diabetic and control subjects (Table 1). As expected [15], AAA1 and Ac-terAA1 were closely correlated with each other in all subjects combined (*r* = 0.550, *p* < 0.001), as well as in T2D patients (*r* = 0.549, *p* < 0.001) and control subjects separately (*r* = 0.542, *p* < 0.001).

Univariate regression analysis showed that in all subjects combined, Ac-terAA1 levels were inversely correlated with total cholesterol, non-HDL cholesterol, triglycerides, apoB, LCAT activity, EST, CET and CEC (Table 2). Similar inverse correlations between these variables and Ac-terAA1 were found in control subjects separately, except for the correlation with CEC, which was not significant (Table 2). These relationships did not reach significance in T2D patients separately. Notably, except for plasma CET, for which the correlation with Ac-terAA1 levels was stronger in control subjects than in T2D patients (*p* for interaction) = 0.025), there were no differences in the strength of the correlations between T2D and control subjects (*p* (interaction) > 0.10 for each). No correlations were observed between both these autoantibodies and glucose, HbA1c, HDL cholesterol, apoA-1, pre-β-HDL formation or CETP mass (Table 2). Of further note, neither in all subjects combined nor in T2D patients and control subjects separately were significant correlations of any of the variables listed in Table 2 with AAA1 demonstrated (Table 2).

In all subjects combined, CEC was positively correlated with pre-β-HDL formation, PLTP and LCAT, as well as with plasma EST and CET in univariate regression analysis (Table 3). Similar relationships were found in control subjects and T2D patients separately. CEC was unrelated to glucose and HbA1c (Table 3). In addition, CEC was positively correlated with non-HDL cholesterol (*r* = 0.450, *p* < 0.001), LDL cholesterol (*r* = 0.286, *p* < 0.001), apoB (*r* = 0.407, *p* < 0.001) and triglycerides (*r* = 0.429, *p* < 0.001). Similar relationships of CE with apoB lipoproteins were found in both groups separately (data not shown).

We next performed multivariable linear regression analysis to disclose the independent associations of CEC with Ac-terAA1 antibodies. This analysis included age, sex, Ac-terAA1 antibodies, and in separate models, also pre-β-HDL formation, PLTP activity, LCAT activity and plasma EST, i.e., variables with which CEC was significantly correlated in univariate analysis. As shown in Table 4, the inverse association between Ac-terAA1 and CEC was unchanged after adjusting for age, sex, diabetes status (model 1) and additionally for the use of glucose-lowering drugs and antihypertensive medication (model 2), as well as after additional adjustment for pre-β-HDL formation and PLTP activity (model 3). However, this association was lost after additional adjustment for plasma EST (Table 4, model 4). These analyses also demonstrated independent and positive associations of CEC with pre-β-HDL formation, PLTP activity and EST.

**Model 1**: adjusted for age, sex and diabetes status.

**Model 2:** adjusted for age, sex, diabetes status, and use of metformin, sulfonylurea and antihypertensive medication.

**Model 3:** adjusted for age; sex; diabetes status; use of metformin, sulfonylurea and antihypertensive medication; and pre-β-HDL formation

**Model 4:** adjusted for age; sex; diabetes status; use of metformin, sulfonylurea and antihypertensive medication; pre-β-HDL formation; and plasma EST

In univariate analysis, there was a strong relationship of plasma EST with CET in all subjects combined (*r* = 0.675, *p* < 0.001), as well as in T2D patients (*r* = 0.695, *p* < 0.001) and control subjects separately (*r* = 0.695, *p* < 0.001). In all subjects combined, there remained a strong relationship of plasma EST with CET after adjustment for age, sex, diabetes status, the use of glucose-lowering drugs and antihypertensive medication, and Ac-terAA1 (β = 0.622, *p* < 0.001; data not shown). Subsequently, we determined the extent to which plasma EST and CET were independently associated with Ac-terAA1. When taking account of age, sex and diabetes status, plasma EST and CET were still inversely and independently associated with Ac-terAA1 levels (Table 5, models 1A and B, respectively). These inverse relationships remained significant after further adjustment for the use of glucose-lowering drugs and antihypertensive medication (Table 5, models 2A and B), and additionally for LCAT activity or CETP mass (Table 5, models 3A and B, respectively). Notably, the associations of plasma EST and CET with Ac-terAA1 were lost after further adjustment for non-HDL cholesterol and triglycerides (Table 5, models 4A and B, respectively).

**Model 1:** adjusted for age, sex and diabetes status.

**Model 2:** adjusted for age, sex, diabetes status, and use of metformin, sulfonylurea and antihypertensive medication.

**Model 3:** adjusted for age; sex; diabetes status; use of metformin, sulfonylurea and antihypertensive medication; and LCAT activity (**A**) or CETP mass (**B**).

**Model 4:** adjusted for age; sex; diabetes status; use of metformin, sulfonylurea and antihypertensive medication; LCAT activity (**A**) or CETP mass (**B**); non-HDL cholesterol; and triglycerides.

## 3. Discussion

The present study is, to our knowledge, the first comprehensive exploration of possible AAA1 and Ac-terAA1 associations with key features of HDL metabolism in T2D patients and control subjects. In line with other reports, T2D patients had unaltered CEC and pre-β-HDL formation, but increased plasma PLTP activity, LCAT activity, EST, CETP mass, CET and triglycerides, together with decreased HDL cholesterol and apoA-1 [27,28,29]. The first remarkable finding of our current study is that Ac-terAA1 and AAA1 do not appear to associate in a similar fashion with parameters of HDL metabolism. In all subjects combined, inverse associations were observed between Ac-terAA1 and total cholesterol, non-HDL cholesterol, triglycerides, apoB, CEC, plasma EST and CET, but these associations were not observed for AAA1. Of note, Ac-terAA1 and AAA1 were not elevated in the diabetic group, and the presence of T2D did in general not significantly modify the associations of CEC and HDL variables with Ac-terAA1. Furthermore, as the opposite of what has been reported before for AAA1 [7,13], no associations between Ac-terAA1, HDL cholesterol and apoA-1 levels were retrieved, a finding which would suggest that Ac-terAA1 may impact on HDL metabolism in a different manner compared to AAA1.

The inverse association between CEC and Ac-terAA1 levels was found to be independent of pre-β-HDL formation, plasma PLTP and LCAT activity, but was lost when taking account of EST. In turn, plasma EST and CET were each inversely correlated with Ac-terAA1, but these autoantibodies were not associated with pre-β-HDL formation and PLTP activity. Remarkably, the associations of Ac-terAA1 with EST and CET were no longer present when adjusting for plasma apoB lipoproteins, particularly triglycerides. Furthermore, CEC was correlated positively with apoB lipoproteins. These results are in accordance with the proposition that CEC, EST and CET are intricately coupled processes, and reiterate the importance of apoB lipoproteins in accepting cholesteryl esters from HDL [30]. Collectively, these results would suggest that Ac-terAA1 may impact on HDL metabolism by influencing plasma cholesterol esterification and cholesteryl ester transfer from HDL towards apoB-containing lipoproteins, without a major effect on ABCA1-mediated efflux via pre-β-HDL and PLTP.

It should be noted that at present there is no gold standard to measure CEC with respect to both the preferred cell system and the acceptor medium. In the current study, we used cholesterol-loaded human cultured fibroblasts as the cholesterol donor and diluted plasma from individual subjects as the cholesterol acceptor medium. Under the experimental conditions employed, these cells abundantly express ABCA1 [31,32]. Lipid-poor HDL particles, i.e., pre-β-HDL, and PLTP are known to stimulate cholesterol efflux by interacting with ABCA1 [31,33,34]. In line with this, CEC was positively related to pre-β-HDL, assayed as the formation of these particles under in vitro conditions of LCAT inhibition, as well as to PLTP activity. Fibroblast also express the ATP-binding cassette transporter G1 (ABCG1), but hardly any scavenger receptor class B type 1 (SR-BI), both of which are reported to interact with mature, spherical HDL particles, although SR-BI is unlikely to mediate net cholesterol mass efflux [33,34,35]. The lack of correlation of CEC with HDL cholesterol and total plasma apoA-1, as shown in the present study, suggests that the CEC assay used here is largely independent of ABCG1. However, the relative contribution of ABCA1, ABCG1 and aqueous diffusion to the efflux process with human cultured fibroblasts is not well known. In addition, though still unsettled, it is relevant to mention that CEC to diluted plasma, which contains pre-β-HDL and other relevant factors involved in cholesterol efflux, could conceivably reflect the interaction of interstitial fluid with cells in a different way compared to isolated HDL. In comparison, using various macrophage cell lines and isolated patient HDL as cholesterol acceptor, CEC did correlate with HDL cholesterol and total plasma apoA-1 [36,37,38,39]. Notably, neither in the present study including diabetic patients with predominantly moderate hyperglycaemia, nor in our recent study evaluating CEC, determined using a macrophage cell line and isolated HDL as cholesterol acceptor, in subjects with various degrees of glucose tolerance, was CEC associated with glycemia. More severely hyperglycaemic circumstances could be required to affect the cholesterol efflux process [40,41].

Although not unequivocally reported, impaired CEC may be associated with accelerated risk of atherosclerosis development, in particular in studies in which macrophage cell lines were used as the cholesterol donor [37,38,42]. Although the use of cultured fibroblasts to determine the association of CEC with incident CVD is unsettled, the inverse relationship of CEC with Ac-terAA1 could imply a proatherogenic role elicited by the presence of these autoantibodies. On the other hand, plasma CET elevations have been shown to predict greater carotid artery intima media thickness and incident CVD [43,44]. Increased plasma EST was also found to confer increased CVD risk, but the role of LCAT per se in atherogenesis is still uncertain [45,46,47,48]. Translating the presently shown relationships between Ac-terAA1 and CEC and EST and CET into CVD risk suggests a mixed picture with possible adverse effects on CEC and beneficial effects on EST and CET.

Several other methodological issues of our study need to be discussed. We excluded subjects using lipid-lowering drugs, since statins may affect HDL metabolism [36]. We also excluded T2D subjects using insulin. This was done to avoid effects of insulin on CEC [49]. As a result, it is likely that diabetic subjects with mild hyperglycaemia and mild dyslipidaemia preferentially participated, explaining modestly elevated fasting glucose and HbA1c, as well as the lack of increase in plasma total cholesterol and non-HDL cholesterol in the participating diabetic patients. This selection may limit the generalizability of our findings. Furthermore, because seropositivity cutoffs for both AAA1 and Ac-terAA1 have not been validated on ethylenediaminetetraacetic acid (EDTA) plasma, we did not perform the analyses according to this strict criterion. Nonetheless, because AAA1 and Ac-terAA1 levels were similar between healthy controls and T2D patients, we would have expected antibody seropositivities close to what have been retrieved in the general population. Furthermore, it is possible that in a larger group of participants, statistically significant relationships of AAA1 and Ac-terAA1 in T2D subjects separately to CEC and HDL metabolism could have been detected. Finally, as a consequence of case-control design of our study, no causality link can be inferred to the associations between Ac-terAA1 and HDL metabolism.

In conclusion, cholesterol efflux capacity, plasma cholesterol esterification and cholesteryl ester transfer are inversely associated with Ac-terAA1, in a way that appears at least in part to be dependent on apoB-containing lipoproteins. The mechanisms responsible for these hitherto unreported associations await further evaluation.

## 4. Materials and Methods

### 4.1. Subjects

The study was approved by the medical ethics committee of the University Medical Centre Groningen, The Netherlands. Subjects from north European descent aged >18 years were recruited by advertisement. All participants provided written informed consent. T2D had been previously diagnosed by primary care physicians using guidelines from the Dutch College of General Practitioners (fasting plasma glucose ≥7.0 mmol/L and/or nonfasting plasma glucose ≥11.1 mmol/L). Diabetic patients who used metformin and/or sulfonylurea were allowed to participate. We excluded patients using insulin or other glucose-lowering drugs. The use of antihypertensive medication and oral contraceptives was allowed. Additional exclusion criteria were clinically manifested cardiovascular disease, renal insufficiency (estimated glomerular filtration rate <60 mL/min/1.73 m^2^ and/or urinary albumin >20 mg/L), liver disease (serum transaminase levels >2 times above the upper reference limit), pregnancy and use of lipid-lowering drugs. Current smokers and subjects who consumed >3 alcoholic drinks daily were also excluded. Physical examination did not reveal cardiac abnormalities. Body mass index (BMI) was calculated as weight (kg) divided by height (m) squared. Blood pressure was measured after 15 min of rest at the left arm using a sphygmomanometer. The participants were evaluated between 8 and 10 h after an overnight fast.

### 4.2. Laboratory Measurements

EDTA-anticoagulated plasma samples were stored at −80 °C until analysis. Plasma glucose was measured shortly after blood collection with an APEC glucose analyser (APEC Inc., Danvers, MA, USA). Glycated haemoglobin (HbA1c) was measured by high-performance liquid chromatography (Bio-Rad, Veenendaal, The Netherlands). Plasma total cholesterol, triglycerides and HDL cholesterol were measured using automated procedures as described. Non-HDL cholesterol was calculated as the difference between total and HDL cholesterol. ApoA-1 and apolipoprotein B (apoB) were assayed by immunoturbidimetry (Roche/Cobas Integra Tina-quant catalog immunoturbidimetry).

Plasma pre-β-HDL formation, i.e., the ability to generate pre-β-HDL, was measured by crossed immunoelectrophoresis [28,50]. Briefly, samples were thawed while kept on ice. Then, 0.9 μmol/L Pefabloc SC (Boehringer-Roche, Penzberg, Germany) and 1.8 μg/L Trasylol (Bayer, Mijdrecht, The Netherlands) were added to inhibit proteolysis (both final concentrations). Samples were incubated during 24 h at 37 °C under conditions of lecithin–cholesterol acyltransferase (LCAT) inhibition, which was achieved by adding iodoacetate (final concentration 1.0 mmol/L) directly after thawing the samples. The crossed immunoelectrophoresis consisted of agarose electrophoresis in the first dimension for separation of lipoproteins with pre-β- and α-mobility. Antigen migration from the first agarose gel into the second agarose gel, containing goat anti-human apoA-1 antiserum, was used to quantitatively precipitate apoA1. The antiserum was monospecific for human apoA-1 using an immunodiffusion assay. Lipoprotein electrophoresis was carried out in 1% (weight/vol) agarose gels in Tris (80 mmol/L)–tricine (24 mmol/L) buffer, 5% (*v*/*v*) polyethylene glycol 300 (pH 8.6) and run in an LKB 2117 system (4 °C for 3 h, 210 V). An amount of 3 μL plasma was added to each well. The track of the first agarose gel was excised and annealed with melted agarose to a gel containing 0.66% (vol/vol) goat anti-human apo A-1 anti-serum (Midland Bioproducts corporation, Boone Iowa) and 0.01% Tween 20 (weight/vol) that was cast on GelBond film (Amersham, Uppsala, Sweden). The plate was run in an LKB 2117 system (4 °C for 20 h, 50 V) in Tris–tricine buffer. Unreacted antibody was removed by extensive washing with saline. The gel was stained with Coomassie Brilliant Blue R250, dried and scanned with a HP Scanjet 5470c. Areas under the pre-β-HDL and α-HDL peaks were calculated. The pre-β-HDL area was expressed as the percentage of the sum of apoA1 in the pre-β-HDL and the α-HDL areas. Pre-β-HDL formation was calculated using the total plasma apoA1 concentration (expressed in apoA-1 (g/L)).

Plasma phospholipid transfer protein (PLTP) activity was assayed with a phospholipid vesicles–HDL system using [^14^C]-labelled dipalmitoyl phosphatidylcholine [27,51]. This method is specific for PLTP activity. PLTP activity is expressed in arbitrary units (AU; 100 AU corresponds to 13.6 μmol phosphatidylcholine transferred per mL/h. Plasma lecithin–cholesterol acyltransferase (LCAT) activity was determined using excess exogenous substrate containing [^3^H]-cholesterol [27]. Briefly, samples were incubated with labelled substrate for 6 h at 37 °C. Corrections were made for the amount of free cholesterol in the samples. The reaction was stopped by adding cold ethanol to the incubation medium. Free and esterified cholesterol were separated using disposable silica columns. [^3^H]-Cholesteryl esters were eluted with hexane. LCAT activity with this assay is strongly correlated with its plasma mass concentration [52,53]. CETP mass was measured using double-antibody sandwich enzyme-linked immunosorbent assays [43]. Plasma CET and EST were determined as described [27,28,29]. To this end, [^3^H]-cholesterol was equilibrated for 24 h at 4 °C with plasma cholesterol, followed by incubation of plasma at 37 °C. Thereafter, apoB-containing lipoproteins were precipitated and the labelled cholesteryl esters were separated from labelled unesterified cholesterol on silica columns. Plasma EST was measured as formation of cholesteryl esters after addition of [^3^H]-cholesterol to plasma using the same incubation system as for the CET assay [27]. CET and EST measurements are expressed in nmol/mL/h.

CEC was determined using human fibroblasts as cholesterol donor [28,29,32,54]. Human skin fibroblasts were obtained from a normolipidemic control subject and were cultured (until passage 5–15) in Dulbecco’s modified Eagle’s medium (DMEM) supplemented with 10% *v*/*v* foetal calf serum in 24-well culture plates to full confluency. After washing them with DMEM, the cells were incubated with [^3^H]-cholesterol (0.5 µCi/ mL) and unlabelled cholesterol (30 µg/mL) for 24 h in order to upregulate ATP-binding cassette transporter A1 (ABCA1) [32]. After cholesterol loading, cells were washed three times with phosphate buffered saline with 0.2% (weight/vol) fatty acid free bovine serum albumin. The efflux assay was carried out by adding 1% plasma diluted in efflux medium and heparin (1.25 U/mL) to prevent clotting. After 4 h incubation at 37 °C, the medium was collected and centrifuged. [^3^H]-Cholesterol was quantified by liquid scintillation counting. Total cellular [^3^H]-cholesterol was determined after extraction of the cells with 2-propanol. CEC was calculated by dividing the radioactive counts in the efflux medium by the sum of the counts in the medium and the cell extract. The CEC assay was performed in duplicate. Values were corrected for appearing in the culture medium in the absence of plasma. To be able to normalise results between series of experiments and to correct for day-to-day variation, efflux to a human plasma pool was determined in quadruplicate. When [^14^C]-cholesterol was added to the plasma-containing efflux medium, the influx rate of [^14^C]-cholesterol from plasma to the cells was consistently <10% over 4 h (triplicate measurements using 3 different plasma samples). Reuptake of newly effluxed labelled cholesterol was, therefore, considered to have no major effect on the CEC measurements.

The inter- and intra-assay coefficients of variation (CVs) of all these assays were <9%.

AAA1and Ac-terAA1 autoantibodies were measured on EDTA plasma using our validated enzyme-linked immunosorbent assay (ELISA) as described previously [2,4,5,6,7,8,9,12,13,14,15]. Maxisorp plates (Nunc™, Roskilde, Denmark) were coated with purified, human delipidated apoA-1 or c-terApoA-1 (20 μg/mL; 50 μL/well) for 1 h (h) at 37 °C. After being washed, all wells were blocked for 1 h with 2% bovine serum albumin (BSA) in a phosphate buffer solution (PBS) at 37 °C. Patient samples were additionally also added to a noncoated well in order to assess individual nonspecific binding. After six washing cycles, 50 μL/well of signal antibody (alkaline phosphatase-conjugated anti-human IgG; Sigma-Aldrich, St Louis, MO, USA), diluted 1:1000 in a PBS/BSA 2% solution, was added and incubated for 1 h at 37 °C. After washing six more times, phosphatase substrate p-nitrophanylphosphate disodium (Sigma-Aldrich) dissolved in a diethanolamine buffer (pH 9.8) was added and incubated for 20 min at 37 °C. Finally, optical density (OD), indicating the immunoreactivity of the antibody, was determined at 405 nm in duplicate (Molecular Devices^TM^ Versa Max). Corresponding nonspecific binding was subtracted from the mean OD for each sample to calculate the net OD of each patient sample, which is expressed in arbitrary units (AU), as previously validated [2,4,5,6,7,8,9,12,13,14,15]. Specificity of our ELISA to detect antibodies against native and lipid-low human apoA1 was previously confirmed by Western blot and tandem-mass spectrometry analyses. For these assays, the interassay CVs were 8.3% and the intra-assay CVs were 4.3%. Given the fact that AAA1 and Ac-terAA1 seropositivity cutoffs have not been determined on plasma EDTA samples, we only considered the continuous immunoreactivities of the antibodies [2,4,5,6,7,8,9,12,13,14,15].

### 4.3. Statistical Analysis

IBM SPSS software (SPSS, version 23.0, Armonk, NY: IBM Corp) was used for data analysis. Because of skewed distribution, natural logarithm (log_e_)-transformed values of triglycerides and the OD values of AAA1 and Ac-terAA1 were used. Results are expressed as mean ± SD for normally distributed variables and as median (interquartile range) for skewed variables. Discrete variables are given as numbers (%). Between groups differences in variables were determined by unpaired t-tests, where appropriate using log_e_ transformed values, or by Chi-square tests. Univariate correlations were determined by Pearson correlation coefficients. Multivariable linear regression analyses were carried out to disclose the independent relationships of CEC, EST and CET with clinical and laboratory variables. Interactions were determined by calculating the product term of Ac-terAA1 OD with the presence of T2D. Two-sided *p*-values < 0.05 were considered significant.

## Figures and Tables

**Table 1:** Clinical and laboratory characteristics of 75 control subjects and 75 Type 2 diabetic patients.

	Control Subjects (*n* = 75)	Type 2 Diabetic Subjects (*n* = 75)	*p*-Value
Age (year)	55 ± 10	59 ± 9	0.014
Sex (M/F)	36/39	47/28	0.10
BMI (kg/m^2^)	25.9 ± 3.9	28.7 ± 4.9	<0.001
Systolic blood pressure (mmHg)	131 ± 20	144 ± 20	<0.001
Diastolic blood pressure (mmHg)	82 ± 11	87 ± 9	<0.001
Plasma glucose (mmol/L)	5.6 ± 0.7	8.8 ± 2.4	<0.001
HbA1c (%)	5.3 ± 0.4	6.7 ± 1.0	<0.001
Total cholesterol (mmol/L)	5.67 ± 0.98	5.40 ± 0.98	0.07
Non-HDL cholesterol (mmol/L)	4.20 ± 1.00	4.12 ± 1.07	0.65
HDL cholesterol (mmol/L)	1.49 ± 0.40	1.28 ± 0.38	0.001
Triglycerides (mmol/L)	1.31 (0.87–1.91)	1.73 (1.17–2.17)	0.036
Apolipoprotein A-1 (g/L)	1.42 ± 0.22	1.28 ± 0.38	0.030
Apolipoprotein B (g/L)	0.95 ± 0.23	0.93 ± 0.23	0.77
CEC (% per 4 h)	8.56 ± 1.00	8.66 ± 0.90	0.52
Pre-β-HDL formation (apoA-1, g/L)	0.31 ± 0.07	0.30 ± 0.07	0.31
PLTP activity (AU)	94.0 ± 10.5	103.6 ± 11.4	<0.001
LCAT activity (AU)	106.8 ± 13.1	114.1 ± 17.4	0.004
EST (nmol/mL/h)	56.1 ± 15.6	63.6 ± 18.9	0.009
CETP mass (mg/L)	2.16 ± 0.67	2.49 ± 0.90	0.011
CET (nmol/mL/h)	20.6 ± 7.5	24.1 ± 9.0	0.011
AAA1(AU)	0.35 (0.24–0.45)	0.27 (0.18–0.39)	0.16
AAc-terAA1 (AU)	0.23 (0.19–0.36)	0.22 (0.15–0.29)	0.17

Data in numbers, mean ± SD or median (interquartile range). AAA1 and Ac-terAA1 autoantibodies are expressed as arbitrary units (AU). BMI, body mass index; CEC, cholesterol efflux capacity; CET, cholesteryl ester transfer; CETP, cholesteryl ester transfer protein; EST, cholesterol esterification; HDL, high-density lipoprotein; LCAT, lecithin–cholesterol acylesterase; PLTP, phospholipid transfer protein. Comparisons are done by unpaired *t*-tests or Chi square analysis. Triglycerides and AUs for AAA1 and Ac-terAA1 autoantibodies are log_e_ transformed.

**Table 2:** Associations of Ac-terAA1 and AAA1, expressed as arbitrary units (AU), with plasma lipids, (apo)lipoproteins, pre-β-HDL, phospholipid transfer protein (PLTP) activity, lecithin–cholesterol acylesterase (LCAT) activity, cholesterol esterification (EST), cholesteryl ester transfer protein (CETP) mass, cholesteryl ester transfer (CET) and cholesterol efflux capacity (CEC) in all subjects combined (**A**) and separately in 75 control subjects (**B**) and in 75 Type 2 diabetic patients (**C**).

**A: All Subjects (*n* = 150)**	**Ac-terAA1 (AU)**	**AAA1 (AU)**
Glucose	−0.080	−0.127
HbA1c	−0.145	−0.019
Total cholesterol	−0.214 ^b^	−0.085
Non-HDL cholesterol	−0.232 ^b^	−0.075
HDL cholesterol	0.078	−0.014
Triglycerides	−0.274 ^c^	−0.122
ApoA-1	0.017	0.001
Apo B	−0.221 ^b^	−0.066
CEC	−0.188 ^a^	−0.126
Pre-β-HDL formation	−0.052	0.030
PLTP activity	−0.072	−0.073
LCAT activity	−0.197 ^a^	−0.077
EST	−0.277 ^c^	−0.093
CETP mass	−0.095	0.062
CET	−0.324 ^c^	−0.093
**B: Control Subjects (*n* = 75)**	**Ac-terAA1 (AU)**	**AAA1 (AU)**
Total cholesterol	−0.298 ^b^	−0.038
Glucose	0.004	−0.193
HbA1c	−0.005	−0.022
Non-HDL cholesterol	−0.352 ^c^	−0.035
HDL cholesterol	0.168	0.108
Triglycerides	−0.449 ^c^	−0.115
ApoA-1	0.100	0.011
Apo B	−0.364 ^c^	−0.042
CEC	−0.l99	−0.115
Pre-β-HDL formation	0.140	−0.003
PLTP activity	−0.177	0.006
LCAT activity	−0.235 ^a^	−0001
EST	−0.414 ^c^	0.015
CETP mass	0.077	0.211
CET	−0.535 ^c^	−0.048
**C: Type 2 Diabetic Subjects (*n* = 75)**	**Ac-terAA1 (AU)**	**AAA1 (AU)**
Glucose	−0.006	−0.043
HbA1c	−0.145	0.135
Total cholesterol	−0.172	−0.179
Non-HDL cholesterol	−0.130	−0.129
HDL cholesterol	−0.078	−0.099
Triglycerides	−0.099	−0.098
ApoA-1	−0.101	−0.054
Apo B	−0.081	−0.101
CEC	−0.165	−0.130
Pre-β-HDL formation	0.022	0.050
PLTP activity	0.022	−0.068
LCAT activity	−0.129	−0.099
EST	−0.131	−0.016
CETP mass	−0.194	−0.016
CET	−0.116	−0.095

Pearson correlation coefficients are shown. Triglycerides and both AAA1 and Ac-terAA1 levels are log_e_ transformed. ^a^ *p* ≤ 0.05; ^b^ *p* ≤ 0.02; ^c^ *p* ≤ 0.01.

**Table 3:** Univariate correlations of cholesterol efflux capacity (CEC) with plasma glucose, HbA1c, pre-β-HDL formation, high-density lipoprotein (HDL) cholesterol, apolipoprotein (apo)A-1, phospholipid transfer protein (PLTP) activity, lecithin–cholesterol acylesterase (LCAT) activity, cholesterol esterification (EST), cholesteryl ester transfer protein (CETP) mass and cholesteryl ester transfer (CET) in all subjects combined (**A**) and separately in 75 control subjects (**B**) and in 75 type 2 diabetic patients (**C**).

**A: All Subjects (*n* = 150)**	**CEC**
Glucose	0.073
HbA1c	0.134
Pre-β-HDL formation	0.283 ^c^
HDL cholesterol	−0.089
ApoA-1	−0.056
PLTP activity	0.313 ^c^
LCAT activity	0.300 ^c^
EST	0.402 ^c^
CETP mass	0.031
CET	0.364 ^c^
**B: Control Subjects (*n* = 75)**	**CEC**
Glucose	0.086
HbA1c	0.032
Pre-β-HDL formation	0.31 ^b^
ApoA-1	−0.005
HDL cholesterol	−0.142
PLTP activity	0.334 ^b^
LCAT activity	0.189
EST	0.320 ^b^
CETP mass	0.004
CET	0.377 ^c^
**C: Type 2 Diabetic Subjects (*n* = 75)**	**CEC**
Glucose	0.052
HbA1c	0.197
Pre-β-HDL formation	0.245 ^a^
ApoA-1	0.140
HDL cholesterol	−0.001
PLTP activity	0.305 ^b^
LCAT activity	0.396 ^c^
EST	0.482 ^c^
CETP mass	0.039
CET	0.352 ^b^

Pearson correlation coefficients are shown. ^a^ *p* ≤ 0.05; ^b^ *p* ≤ 0.01; ^c^ *p* ≤ 0.01.

**Table 4:** Multivariable linear regression analysis showing the association of Ac-terAA1, expressed as optical density, with cholesterol efflux capacity in 150 subjects.

	Model 1		Model 2		Model 3		Model 4	
	β	*p*-value	β	*p*-value	β	*p*-value	β	*p*-value
Age	−0.026	0.75	−0.068	0.43	−0.121	0.13	−0.098	0.20
Sex (male vs. female)	0.157	0.23	−0.063	0.46	0.027	0.74	0.019	0.81
T2D	0.053	0.53	−0.025	0.84	−0.030	0.80	−0.027	0.81
Ac-terAA1 (AU)	−0.178	0.032	−0.186	0.026	−0.157	0.044	−0.088	0.26
Pre-β-HDL formation					0.282	0.001	0.240	0.003
PLTP activity					0.245	0.007	0.185	0.003
EST							0.288	0.038

β: standardized regression coefficient. EST, cholesterol esterification; AU, arbitrary units; PLTP, phospholipid transfer protein; T2D, Type 2 diabetes mellitus. Ac-terAA1 levels are log_e_ transformed.

**Table 5:** Multivariable linear regression analysis showing the association of Ac-terAA1 with plasma cholesterol esterification (EST) (**A**) and plasma cholesteryl ester transfer (CET) (**B**) in 150 subjects.

**A: EST**	**Model 1**		**Model 2**		**Model 3**		**Model 4**	
	β	*p*-value	β	*p*-value	β	*p*-value	β	*p*-value
Age	−0.058	0.47	−0.049	0.54	−0.037	0.57	0.013	0.08
Sex (male vs. female)	−0.057	0.48	−0.034	0.67	−0.007	0.91	−0.065	0.26
T2D	0.205	0.012	0.010	0.93	−0.033	0.72	−0.001	0.99
Ac-terAA1 (AU)	−0.252	0.002	−0.261	0.001	−0.164	0.012	−0.071	0.22
LCAT activity					0.566	<0.001	0.270	0.001
Non-HDL cholesterol							−0.029	0.70
Triglycerides							0.522	<0.001
**B: CET**	**Model 1**		**Model 2**		**Model 3**		**Model 4**	
	β	*p*-value	β	*p*-value	β	*p*-value	β	*p*-value
Age	−0.158	0.045	−0.163	0.036	−0.154	0.045	−0.085	0.074
Sex (male vs. female)	0.067	0.39	0.095	0.21	0.120	0.12	0.031	0.97
T2D	0.195	0.015	−0.035	0.75	−0.089	0.41	0.003	0.97
Ac-terAA1 (AU)	−0.308	<0.001	−0.321	<0.001	−0.312	<0.001	−0.087	0.077
CETP mass					0.207	0.008	0.141	0.004
Non-HDL cholesterol							0.313	<0.001
Triglycerides							0.506	<0.001

β: standardized regression coefficient. CET, cholesteryl ester transfer; CETP, cholesteryl ester transfer protein; EST, cholesterol esterification; LCAT, lecithin–cholesterol acyltransferase; non-HDL, non-high-density lipoproteins; AU, arbitrary units; T2D, Type 2 diabetes mellitus. Ac-terAA1 and triglyceride levels are log_e_ transformed.

## Abbreviations

AAA1	autoantibodies against apolipoprotein A-1
ABCA1	ATP-binding cassette transporter A1
ABCG1	ATP-binding cassette transporter G1
Ac-terAA1	autoantibodies against c-terminus of apolipoprotein A-1
Apo	apolipoprotein
AU	arbitrary units
BMI	body mass index
BSA	bovine serum albumin
CEC	cholesterol efflux capacity
CV	coefficient of variation
CET	cholesteryl ester transfer
CETP	cholesteryl ester transfer protein
DMEM	Dulbecco’s modified Eagle’s medium
ELISA	enzyme-linked immunosorbent assay
EDTA	ethylenediaminetetraacetic acid
EST	cholesterol esterification
HDL	high-density lipoproteins
LCAT	lecithin–cholesterol acylesterase
OD	optical density
PBS	phosphate buffer solution
PLTP	phospholipid transfer protein
T2D	Type 2 diabetes mellitus
